# Comparative early outcomes following primary radiofrequency ablation and polidocanol microfoam ablation of symptomatic, incompetent small saphenous veins

**DOI:** 10.1016/j.jvsv.2025.102234

**Published:** 2025-03-20

**Authors:** Mokhshan Ramachandran, Peter F. Lawrence, Steven M. Farley, David A. Rigberg, Johnathon Rollo, Vincent L. Rowe, Juan Carlos Jimenez

**Affiliations:** Division of Vascular and Endovascular Surgery, Gonda Venous Center, David Geffen School of Medicine at UCLA, Los Angeles, CA

**Keywords:** Venous, Insufficiency, Varicose, Ulceration, Edema, Microfoam

## Abstract

**Background:**

Radiofrequency ablation (RFA) of symptomatic, incompetent small saphenous veins (SSVs) is supported by clinical practice guidelines, but polidocanol microfoam ablation (MFA) is not addressed in these guidelines owing to the absence of high-quality clinical data. However, some anatomical variations and clinical scenarios in patients with SSV reflux may be associated with equivalent or superior results when MFA is used compared with RFA. This study aims to compare early outcomes after the treatment of SSV incompetence in patients with Clinical-Etiology-Anatomy-Pathophysiology (CEAP) 2 class to 6 disease using either RFA or MFA.

**Methods:**

A retrospective review of a prospectively maintained database was conducted among patients who underwent treatment of incompetent SSVs with either RFA or MFA. Limbs that underwent concomitant phlebectomy were included. All patients underwent postoperative duplex ultrasound at 48 to 72 hours and at least one follow-up visit by a vascular surgery provider. Primary outcomes were immediate SSV closure and ablation-related thrombus extension. Secondary outcomes analyzed included demographic data, CEAP clinical class, Venous Clinical Severity Score (VCSS), deep venous thrombosis, and adverse events.

**Results:**

Between March 2018 and July 2024, 182 SSVs treated for symptomatic reflux with either RFA (n = 120) or MFA (n = 62) were identified. Age, gender, body mass index, reflux times, and SSV diameters were similar between both groups. The mean preoperative VCSSs were 9.4 ± 3.0 and 10.8 ± 3.7 in the RFA and MFA groups, respectively (*P* = .05). More venous ulcers were present at the time of MFA (n = 16 [26%]) than RFA (n = 14 [12%]) (*P* = .015). Median follow-up was 164.5 days in the RFA cohort and 156 days after MFA. Symptomatic improvement after RFA and MFA was 91% and 88%, respectively. The mean postoperative VCSS decreased from 9.4 to 7.3 in the RFA group (*P* < .001) and from 10.9 to 9.2 after MFA (*P* < .001). Immediate vein closure was achieved in 98% of limbs in both groups; two late recanalizations occurred after MFA, but none after RFA. The number of ulcers healed at last follow-up was greater after MFA (n = 13 [81%] vs n = 10 [71%]; *P* = .02). The incidence of ablation-related thrombus extension was 4.8% (n = 3) after MFA and 1.7% (n = 2) after RFA (*P* = .52). One gastrocnemius deep venous thrombosis occurred in the MFA group. No pulmonary emboli or central nervous complications occurred. All adverse thrombotic events were asymptomatic and resolved with short-term anticoagulation. Superficial phlebitis was higher after MFA (n = 11 [17.7%] vs n = 5 [4.2%]; *P* = .002) One postoperative sural neuralgia occurred after RFA.

**Conclusions:**

RFA and MFA are both safe and effective treatments for patients with symptomatic, incompetent SSVs. Both resulted in excellent clinical relief and early truncal vein closure rates. The number of ulcers healed was higher in the MFA group, but this difference was significant on univariate analysis only. Adverse thrombotic events after RFA were low and consistent with other contemporary studies, although superficial phlebitis was more frequent after MFA.

**Article Highlights**

• **Type of Research:** Single-center retrospective cohort study
• **Key Findings:** We treated 182 small saphenous veins with either radiofrequency ablation (RFA) (n = 120) or microfoam ablation (MFA) (n = 62) for symptomatic reflux. Clinical improvement after RFA and MFA was 91% and 88%, respectively. Early vein closure was achieved in 98% of limbs in both groups. More ulcers healed after MFA (n = 13 [81%] vs n = 10 [71%]; *P* = .02) although associated with higher rates of superficial phlebitis (MFA 17.7% vs 4.2%; *P* = .002).
• **Take Home Message:** RFA and MFA are both safe and effective treatments for patients with symptomatic, incompetent small saphenous veins. Both resulted in excellent clinical relief and early truncal vein closure rates. More ulcers healed in the MFA group, but with higher rates of superficial phlebitis.

Radiofrequency ablation (RFA) for the treatment of symptomatic SSV reflux has been extensively studied and validated in the peer-reviewed literature as safe and effective.^1^, ^2^, ^3^, ^4^ The most recent consensus guidelines from the Society for Vascular Surgery, American Venous Forum, and the American Vein and Lymphatic Society recommend RFA from the knee to the upper or mid-calf over physician-compounded ultrasound-guided foam sclerotherapy based on low-quality (Grade 2C) evidence.^5^ Because fewer comparative studies are available and the overall quality of published evidence is lower compared with the great saphenous vein (GSV), a gold standard modality for the treatment of the incompetent SSV is less well-defined.^6^

The SSV is believed to be more anatomically variable than the GSV and certain anatomical features, such as tortuosity, can make passage of an RFA catheter through the vein difficult.^7^ Additionally, the sural nerve lies in closer proximity to the SSV in the distal leg; thus, neuralgia after endovenous thermal ablation is possible.^8^ Although reported rates are low, sural neuralgia after thermal ablation can be functionally limiting.^9^

Nonthermal modalities have emerged that expand the options for treating the SSV below the knee. Commercially manufactured polidocanol microfoam (Varithena, Boston Scientific, Marlborough, MA) was approved by the US Food and Drug Administration in 2013. It is indicated for the treatment of incompetent GSVs and anterior saphenous veins, as well as visible varicosities of the GSV system above and below the knee. Its use in the SSV is considered off label owing to insufficient evidence in this anatomical location. One potential advantage is that thermal sural nerve injury is eliminated with MFA because heat is not used. Another is microfoam's ability to travel intraluminally through tortuous anatomy and superficial veins within the subulcer plexus in limbs with venous stasis wounds. Comparative studies analyzing the efficacy and safety of RFA vs MFA for the treatment of symptomatic, incompetent SSVs are currently lacking. Our study analyzes and compares outcomes after the treatment of symptomatic, incompetent SSVs with both RFA and MFA.

## Methods

We retrospectively reviewed a prospectively maintained patient database for all procedures performed in our venous ambulatory procedure unit (APU). Our study was approved by the institutional review board, and the need for patient consent was waived. Our APU is affiliated with a large, urban tertiary care academic medical center. All patients who underwent RFA and MFA for symptomatic SSV and tributary reflux between March 2018 and July 2024 were identified.

Symptomatic patients (C2(s)-C6(s)) were included and presented with lifestyle-limiting physical signs and symptoms. Specific symptoms included pain, heaviness, fatigue, leg swelling, venous stasis skin changes (lipodermatosclerosis), and venous ulcers. To meet inclusion criteria, all limbs required duplex ultrasound findings of reflux ≥0.5 seconds occurring at the saphenopopliteal junction and in the immediately caudal SSV in the standing position. Before SSV closure, a failed period of conservative therapy was required with 4 to 6 weeks of compression stocking use (minimum pressure, 20-30 mm Hg). All patients with venous ulceration were treated concomitantly with regular dressing changes, offloading, and compression therapy.

Beginning in March of 2018, all consecutive limbs treated with MFA of the SSV were identified. All consecutive limbs treated with RFA of the SSV meeting study criteria during the same period were identified and used as a comparison group. Limbs treated with concomitant phlebectomy were also included in the cohort. Our procedural techniques used for both MFA and RFA have been described previously.^10^^,^^11^

Primary outcomes were immediate SSV closure and ablation-related thrombus extension (ARTE). Secondary pre-procedure variables reviewed included age, gender, body mass index, concomitant deep venous reflux, SSV reflux times, SSV maximal diameter, prior proximal GSV ablation, history of bleeding, prior deep venous thrombosis (DVT), chronic oral anticoagulation, Clinical-Etiology-Anatomy-Pathophysiology (CEAP) clinical class, and Venous Clinical Severity Scores (VCSS). Secondary procedural variables measured included operative times, the volume of microfoam used per session, and the performance of concomitant phlebectomy.

All limbs in this study had a duplex ultrasound performed within 48 to 72 hours after the -procedure to evaluate SSV closure and rule out ARTE and DVT. Patients were counseled to wear 20- to 30-mm Hg compression stockings 14 days after their procedure. All patients in this cohort had at least one follow-up visit with a vascular surgery provider approximately 3 to 6 weeks after their SSV closure. Patients who did not undergo a postprocedure ultrasound at 48 to 72 hours and at least one follow-up visit were excluded. After this early postprocedure ultrasound, subsequent imaging was based on surgeon preference. It was not routinely performed unless the patient presented with leg symptoms (ie, pain, phlebitis, edema, recurrent veins), incomplete closure, ARTE, or DVT during the study period. After the initial follow-up office visit, patients continued return office visits based on the individual surgeon's preference.

Secondary postprocedure variables analyzed included leg pain, superficial thrombophlebitis, DVT, requirement for new anticoagulation, change in VCSS, symptomatic improvement, and the proportion of ulcers healed. The determination of postprocedural pain and swelling was subjectively determined by history and physical examination by the examining provider. VCSSs were calculated at the patient's last documented follow-up visit.

## Statistical analysis

Qualitative variables were reported in the form of numbers or proportions, whereas quantitative variables were reported in the form of means ± standard deviation, in accordance with standard statistical methods. We analyzed baseline demographic variables between the two procedural groups (RFA and MFA) using Pearson's χ^2^ test for the analysis of qualitative variables and Student's *t* test for the comparison of quantitative variables. Additionally, pertinent perioperative variables, including SSV reflux time, vein diameter, and pre-/post-treatment VCSS scores were analyzed and reported in a similar fashion (Table I). Univariate analysis was implemented for the comparison of MFA and RFA for the following outcome variables: ARTE, ARTE/DVT, vein nonclosure, the requirement of new postoperative anticoagulation, postprocedure pain, swelling or phlebitis, healed ulcer, and symptomatic improvement. A multivariate logistic regression model was used to analyze variables that were significant in univariate analysis. These included concomitant phlebectomy, the presence of an ulcer at the time of treatment, and a history of bleeding. Multivariate analysis using logistic regression for primary outcomes by procedure type adjusted for age, gender, body mass index, prior ablation, concomitant phlebectomy, and bleeding history was also performed.

**Table I:** Univariate analysis: Baseline characteristics of 182 patients receiving treatment for small saphenous vein insufficiency by procedure type

	RFA (n = 120)	Varithena (n = 62)	*P* value
Age, years	61.14 ± 13.74	64.97 ± 15.45	.278
Gender			.120
Male	53 (44.2)	20 (32.3)	
Female	67 (55.8)	42 (67.7)	
BMI	27.85 ± 6.49	27.34 ± 5.58	.185
Operative time, minutes	43.03 ± 16.50	34.90 ± 16.54	.982
Deep venous reflux	70 (58.3)	39 (62.9)	.551
SSV reflux time, minutes	3.31 ± 1.46	3.23 ± 1.46	.979
SSV max diameter, mm	6.26 ± 2.28	5.29 ± 2.10	.465
Prior proximal GSV ablation	50 (41.7)	40 (64.5)	**.003**
Concomitant stab phlebectomies	37 (30.8)	7 (11.3)	**.004**
Pretreatment VCSS score	9.35 ± 2.96	10.87 ± 3.66	.054
Post-treatment VCSS score at last follow-up	7.29 ± 2.47	9.18 ± 3.46	**.002**
Ulcer at time of treatment	14 (11.7)	16 (25.8)	**.015**
Prior DVT	8 (6.7)	6 (9.7)	.470
History of bleeding	1 (0.8)	6 (9.7)	**.003**
Chronic anticoagulation	24 (20.0)	8 (12.9)	.233

*BMI,* Body mass index; *DVT,* deep vein thrombosis; *GSV,* great saphenous vein; *SSV,* small saphenous vein; *VCSS,* venous clinical severity score.

Values are mean ± standard deviation or number (%).

Boldface entries indicate statistical significance.

All analytical methods were executed using Stata 18.0 software (StataCorp LLC, College Station, TX).

## Results

Between March 2018 and July 2024, 1563 saphenous vein ablations (1054 RFA and 509 MFA) were performed in our venous APU. Sixty-two SSVs in 53 patients were treated with MFA, and all met the study inclusion criteria. During the same period, 154 total SSV RFAs were performed, and 120 in 101 patients met the study inclusion criteria. These procedures were used as a comparison group. The decision to treat the SSV primarily was based on the surgeons' preference. Comparative preprocedure patient demographics and variables are provided in Table I. Multivariate analysis of significant univariate predictors can be found in Table II. A summary of limbs treated in the study cohort by CEAP clinical class can be found in the Fig. Age, gender, and body mass indices were similar in both groups. The mean VCSS before treatment was 9.4 ± 3.0 and 10.8 ± 3.7 in the RFA and MFA groups, respectively. Concomitant deep vein reflux was present in 58% (RFA) and 63% (MFA). Mean maximal SSV diameters in the RFA and MFA groups were 6.3 ± 2.3 mm and 5.3 ± 2.1 mm, respectively (*P* = .47). More patients in the MFA group (26%) had a venous ulcer at the time of SSV closure than in the RFA group (12%) (*P* = .015). All ulcers in this study cohort were in the medial and lateral leg below the knee.

**Table II:** Multivariate analysis of significant univariate predictors

Variable	Significant univariate predictors			Multivariate analysis	
	RFA	Varithena	*P* value	OR (95% CI)	*P* value
Concomitant phlebectomies	37 (30.8)	7 (11.3)	.004	0.26 (0.1-0.67)	**.001**
Ulcer at time of treatment	14 (11.7)	16 (25.8)	.015	3.03 (1.19-7.68)	**.001**
History of bleeding	1 (0.8)	6 (9.7)	.003	12.75 (1.50-108.43)	.**003**

*CI,* Confidence interval; *OR,* odds ratio; *RFA,* radiofrequency ablation.

Values are number (%) unless otherwise indicated.

Boldface entries indicate statistical significance.

**Fig:**
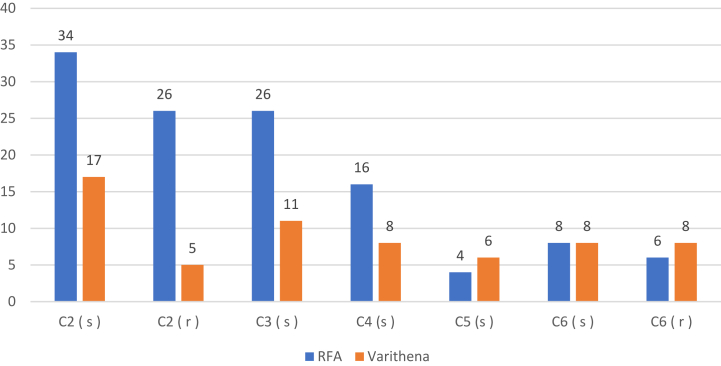
Summary of limbs treated in the study by CEAP clinical class. *CEAP*, Clinical-Etiology-Anatomy-Pathophysiology; *(r)*, recurrent active venous ulcer; *RFA*, radiofrequency ablation; *(s)*, symptomatic.

The mean operative times were similar in both groups (RFA, 43.0 ± 16.5 minutes; MFA, 35.0 ± 16.5 minutes; *P* = .98). Concomitant phlebectomies were performed during 31% of RFAs and 11% of MFAs (*P* = .004). None of the patients who underwent RFA of the SSV to the midcalf underwent concomitant sclerotherapy. The average volume of microfoam used was 4.5 ± 2.3 mL per MFA procedure.

A summary of univariate and multivariate analysis for outcomes by treatment type can be found in Table III. The median follow-up in the RFA group was 164.5 days (interquartile range, 14-2280 days) and 156 days (interquartile range, 15-2040 days) after MFA (*P* = .98). The treated SSV immediately closed in 98% of limbs in both groups (RFA, n = 118; MFA, n = 61). One SSV remained patent after MFA at the first postprocedure duplex ultrasound examination and later closed after RFA. Two treated veins that were confirmed closed by duplex immediately after MFA recanalized during the study period (at 14 and 240 days). One of these patients (recanalized at 14 days) was newly started on oral anticoagulation. The other underwent subsequent ligation and partial stripping with clinical improvement. Two SSVs remained patent immediately after RFA. One partially closed 4 months later as noted by duplex ultrasound examination. Both opted for no additional treatment. The incidence of SSV nonclosure was not statistically different between the groups (*P* = .98).

**Table III:** Univariate and multivariate analysis for primary outcomes by procedure type

Variable	Univariate analysis			Multivariate analysis	
	RFA	Varithena	*P* value	OR (95% CI)	*P* value
ARTE	2 (1.7)	3 (4.8)	.215	1.87 (0.28-12.52)	.287
ARTE/DVT	2 (1.7)	4 (6.5)	.087	4.07 (0.72-22.87)	.098
Vein nonclosure	2 (1.7)	1 (1.6)	.978	0.97 (0.09-10.88)	.978
Required new postoperative AC	2 (1.7)	4 (6.5)	.087	4.07 (0.72-22.87)	.098
Postprocedure pain	9 (7.5)	9 (14.5)	.133	2.97 (1.04-8.47)	**.042**
Postprocedure phlebitis	5 (4.2)	11 (17.7)	.002	4.26 (1.32-13.77)	**.013**
Postprocedure swelling	2 (1.7)	1 (1.6)	.978	1.18 (0.10-13.37)	.893
Ulcer healed	10 (8.3)	13 (21.0)	0015	1.33 (0.22-7.98)	.752
Symptomatic improvement	109 (90.8)	55 (87.1)	.649	1.16 (0.41-3.28)	.316

*AC,* Anticoagulation; *ARTE,* ablation-related thrombus extension; *CI,* Confidence interval; *DVT,* deep vein thrombosis; *OR,* odds ratio; *RFA,* radiofrequency ablation.

Values are number (%) unless otherwise indicated.

Boldface entries indicate statistical significance.

The mean VCSS decreased to 7.3 ± 2.5 (*P* < .001) and 9.2 ± 3.5 (*P* < .001) in the RFA and MFA groups, respectively. Clinical improvement was reported at the last follow-up for 91% after RFA and 89% after MFA (*P* = .65). The absolute ulcer healing rates during the study period were superior in the MFA group (81%; 13 of 16 healed) compared with RFA (71%; 10 of 14 healed). This difference was statistically significant in univariate (*P* = .02) but not multivariate (*P* = .752) analysis. The proportion of healed recurrent ulcers in the MFA group was 75% (6 of 8 healed) and 50% (3 of 6 healed) in the RFA group and was not statistically different (*P* = .33). Symptomatic superficial thrombophlebitis in tributary veins occurred more frequently after MFA (RFA, n = 5 vs MFA, n = 11; *P* = .002). Nonspecific postprocedure pain (RFA, n = 9 vs MFA, n = 9; *P* = .133) and swelling (RFA, n = 2 vs MFA, n = 1; *P* = .978) were similar in both groups on univariate analysis. One patient developed transient paresthesia along the sural nerve distribution after RFA that resolved at one month. No skin burns occurred.

Overall thrombotic complications in the study are summarized in (Tables IV and V). ARTE into the popliteal vein occurred in two limbs after RFA (1.7%) and 3 after MFA (4.8%) (*P* = .22). All were asymptomatic and detected on early postprocedure ultrasound. One asymptomatic gastrocnemius DVT occurred after MFA. No remote DVT was noted after RFA. All patients in this cohort with ARTE and DVT were treated with oral apixaban (5 mg bid) for a median duration of 13.5 days (interquartile range, 3-65 days) and resolved on follow-up duplex examination. No symptomatic DVT or pulmonary emboli occurred in either group.

**Table IV:** Characteristics of patients with ablation-related thrombus extension (*ARTE*) and deep venous thrombosis (*DVT*) after microfoam ablation (MFA)

MFA	Thrombotic event	Max SSV diameter, mm	CEAP clinical class	Volume of microfoam used, mL	BMI	Operative time, minutes	History of prior DVT	Duration of anticoagulation, days	Level	Resolved
Patient A	ARTE popliteal vein	4.9	2	3	22	51	No	7	II	Yes
Patient B	ARTE popliteal vein	5.2	2	3	27	47	No	65	II	Yes
Patient C	ARTE POPLITEAL Vein	5.9	5	3	31	40	No	17	II	Yes
Patient D	Gastrocnemius DVT	3.1	2	5	24	67	No	32	NA	Yes

*BMI,* Body mass index; *CEAP,* clinical-etiology-anatomy-pathophysiology; *SSV,* small saphenous vein.

**Table V:** Characteristics of patients with ablation-related thrombus extension (*ARTE*) after radiofrequency ablation (*RFA*)

RFA	Thrombotic event	Max SSV diameter, mm	CEAP clinical class	BMI	Operative time, minutes	History of prior DVT	Duration of anticoagulation, days	Level	Resolved
Patient E	ARTE popliteal vein	7.4	6	57	37	No	3	II	Yes
Patient F	ARTE popliteal vein	9.5	4	25	20	No	10	III	Yes

*BMI,* Body mass index; *CEAP,* clinical-etiology-anatomy-pathophysiology; *DVT,* deep venous thrombosis; *MFA,* microfoam ablation; *SSV,* small saphenous vein.

## Discussion

This study is the first published comparative analysis between RFA and commercially manufactured microfoam ablation (MFA) for patients with symptomatic SSV incompetence. Treatment of SSV reflux with commercially manufactured endovenous polidocanol microfoam was not included in phase 3 randomized trials, which led to US Food and Drug Administration approval for use in the great and anterior saphenous veins.^12^, ^13^, ^14^ Although there is limited evidence that MFA can result in good early closure rates in the SSV, there is limited comparative data between thermal techniques and Varithena for treatment in this anatomical location, where its use remains off label.^15^

Our study demonstrated high early closure rates after both RFA and MFA, consistent with prior published reports for the GSV.^16^^,^^17^ Both modalities also demonstrated improved VCSSs after treatment and overall clinical improvement at the last follow-up with no statistical difference. Superficial thrombophlebitis in the SSV and associated tributaries occurred more frequently after MFA than RFA. This finding is consistent with prior published findings for the GSV and anterior saphenous veins. A possible mechanism is more widespread endothelial contact of the microfoam with superficial SSV tributaries, leading to an inflammatory response. In our study, all episodes of phlebitis resolved without long-term clinical sequelae in both groups.

Endovenous MFA demonstrated an advantage in patients with venous ulcers, with an increased absolute ulcer healing rate compared with RFA. His difference was significant in univariate but not multivariate analysis. This is consistent with prior studies in the GSV published from our institution.^16^ A recent study by Zhu et al^18^ demonstrated a 100% ulcer healing rate in 42 limbs treated with ultrasound and fluoroscopy-guided foam sclerotherapy. Although their study was conducted with physician-compounded foam and not Varithena, the authors proposed that infiltrating incompetent feeding veins and surrounding veins in the subcutaneous venous plexus may improve ulcer healing.^18^ Because RFA cannot directly target these vessels, this mechanism may explain the higher ulcer healing rates in the MFA group in our current study. The 2022 European Society for Vascular Surgery Clinical Practice Guidelines recommend that ablation of the subulcer plexus with ultrasound-guided foam sclerotherapy be considered part of the treatment strategy for venous ulcers.^19^

The incidence of ARTE after RFA was low (1.7%) and consistent with the peer-reviewed literature. Current guidelines recommend foregoing the routine use of post-RFA duplex ultrasound examination in asymptomatic patients because of low associated rates of thrombotic complications. Although the difference in rates of ARTE in this study did not reach statistical significance between the two modalities, the overall rate of ARTE (4.8%) after MFA suggests that postprocedure duplex ultrasonography still plays an important role when this treatment is used. Routine early post-MFA ultrasound should still be performed until further studies are able to determine clearly the natural history of ARTE after MFA. Using the surveillance protocol described in our study, all patients who experienced thrombotic events were asymptomatic and resolved without long-term morbidity with a short course of oral apixaban.

Limitations include a nonrandomized, retrospective protocol, increasing selection bias risk. Owing to the limited numbers in our cohort, propensity score matching was not used. Because of the variable follow-up and lack of long-term duplex screening, longer-term closure rates between modalities cannot be assessed and are the focus of future studies. Structured quality of life metrics such as the Aberdeen Questionnaire and VEINES-QOL/SYM (Venous Insufficiency Epidemiological and Economic Study-Quality of Life/Symptoms) were not used. The duration of ulcers before presentation at our institution and image analysis software data tracking ulcer healing rates were also not available. The rates of deep venous reflux were high in both treatment groups. Thus, it is likely that patients with secondary disease were included. VCSSs in patients with secondary disease may not be expected to change to a similar degree after SSV ablation as patients with solely primary disease. Additionally, there was an unequal distribution of RFA and MFA cases among the treating surgeons. Two surgeons (of the six) performed the majority of the microfoam cases. This was based on personal preference and experience.

## Conclusions

Both RFA and MFA are effective and safe treatments for symptomatic, refluxing SSVs. They provide excellent clinical relief and high early closure rates. MFA showed a greater proportion of ulcers healed compared with RFA. This result was significant in univariate but not multivariate analysis. The rate of adverse thrombotic events (ARTE and DVT) after RFA is low and consistent with findings from other contemporary studies. MFA is associated with a greater frequency of superficial phlebitis compared with RFA. Although rates of ARTE were not statistically different between the two groups, we recommend the continued use of early duplex surveillance with selective anticoagulation until the natural history of ARTE after MFA is better defined in the peer-reviewed literature.

## Author contributions

Conception and design: SF, DR, JJ

Analysis and interpretation: MR, PL, JR, JJ

Data collection: MR, JJ

Writing the article: MR, PL, SF, DR, JR, JJ

Critical revision of the article: MR, PL, SF, DR, JR, JJ

Final approval of the article: MR, PL, SF, DR, JR, JJ

Statistical analysis: MR

Obtained funding: Not applicable

Overall responsibility: JJ

## Funding

None.

## Disclosures

J.C.J. is a consultant for Boston Scientific. J.R. is a consultant for Boston Scientific.
